# Supports Used by Aboriginal and Torres Strait Islander Women for Their Health, including Smoking Cessation, and a Baby’s Health: A Cross-Sectional Survey in New South Wales, Australia

**DOI:** 10.3390/ijerph17217766

**Published:** 2020-10-23

**Authors:** Gillian S. Gould, Carl Holder, Christopher Oldmeadow, Maree Gruppetta

**Affiliations:** 1 School of Medicine and Public Health, The University of Newcastle, Callaghan 2308, Australia; Maree.Gruppetta@newcastle.edu.au; 2 Hunter Medical Research Institute, New Lambton Heights 2305, Australia; ctah78_work@hotmail.com (C.H.); Christopher.Oldmeadow@hmri.org.au (C.O.)

**Keywords:** Aboriginal women, Torres Strait Islander women, smoking cessation, pregnancy, child health, maternal health, prenatal health, health services, online support

## Abstract

This study explored Aboriginal and Torres Strait Islander women’s use of supports for their general health, for smoking cessation, and the health of babies or children, and analyzed the women’s predictors for seeking types of support. Aboriginal and Torres Strait Islander women were recruited for a cross-sectional survey in two regions of NSW *N* = 132. The 19-item survey questioned the likelihood that the participant would use the various supports for their health, to quit smoking, and for a baby or child’s health. Logistic regression analyses were performed on *N* = 98 with complete data. Older participants were less likely to use Facebook or the internet for their health, or the health of a child, but were more likely to consult with health professionals. Women who had quit smoking were less likely to use an app for their health compared to smokers. Women who had a child living in their household were less likely to use the internet for a child’s health. This community-based study revealed age-related differences for access to health services and differences according to smoking status. Patterns of internet and app use warrant further consideration when planning strategies to improve Aboriginal and Torres Strait Islander women and children’s health.

## 1. Introduction

Closing the Gap strategic priorities emphasize the importance of improving the health of Aboriginal and Torres Strait Islander people. One of these priorities centers around improving the birth weight of babies born to Indigenous parents. Several factors may impact on a baby’s birth weight, but recent studies have indicated the lower birth weight, seen among Aboriginal and Torres Strait Islander babies, is mainly accounted for by tobacco use in pregnancy [1]. It must be acknowledged that continued tobacco use among Aboriginal and Torres Strait Islander people in Australia is a corollary of the social determinants of health [2,3], historical factors imposed by past and ongoing colonization, and racism [4].

In Australia, the predominantly used avenues for Aboriginal and Torres Strait Islander people seeking help to quit smoking are through primary health services [5]. Services include Aboriginal Community Controlled Health Services (ACCHS), general practices, and other primary health services. National strategies such as the Tackling Indigenous Smoking program include currently 37 regional teams and the state Quitlines [6]. Pregnant women may also access smoking cessation care via maternity services such as antenatal clinics, and in some states such as New South Wales (NSW), specialized clinics are provided for Aboriginal and Torres Strait Islander women [7]. Globally and in Australia, specifically, service provision gaps have been highlighted for smoking cessation care directed to pregnant women [8,9]. Gaps include a lack of training among health providers and low access to suitable forms of counselling and pharmacotherapies [10,11].

Apart from limited information about how Aboriginal and Torres Strait Islander people seek help from health services [5,12,13], there is little known about which avenues are used by Aboriginal and Torres Strait Islander women for seeking health more directly. These avenues might include ways to receive information on their own health or as carers, and information about a baby or child’s health. If a woman or her significant others smoke, where would she go to get help to quit smoking? Options may include seeking help from friends, family and community groups, or the use of digital resources such as the internet, social media sites, e.g., Facebook, mobile applications, or by telephone. There is little guidance for those developing such additional resources when little is known about how a target audience may be segmented, i.e., which sub-groups may be more likely to access certain avenues for health-seeking.

This study aimed to survey Aboriginal and Torres Strait Islander women to (1) assess their use of a variety of supports for their overall health and more specifically for smoking cessation, and also for the health of babies or children; (2) analyze the impact of a variety of predictors on health-seeking for self, smoking, and baby via various avenues for support. The avenues considered were health professionals, friends, family, community groups, digital resources, i.e., the internet, Facebook, mobile applications, and telephone. This information may be useful in developing strategies for different sub-groups for social marketing.

## 2. Materials and Methods

### 2.1. Methods

A cross-sectional survey was administered in two regions of NSW: the Mid North Coast (MNC) and Hunter New England (HNE). Specific communities on the MNC were Coffs Harbour and Kempsey, and in HNE: Armidale, Tingha, Inverell and Glen Innes. In total *N* = 137 surveys were completed; however, five women were later found to be ineligible.

Women were eligible to be in the study if they were 16 years old or over and were able to give informed consent (*N* = 132). The majority of surveys (*n* = 79) were conducted before 11 pre-arranged women’s focus groups from February to March 2018. In addition, a further (*n* = 53) women were approached by personal intercept by an Aboriginal Research Assistant at a National Aborigines and Islanders Day Observance Committee (NAIDOC) event in Coffs Harbour in July 2018. The sample was a convenience sample, but attempts were made to purposively obtain a range of views from Aboriginal women of different age groups, pregnant and non-pregnant, those who smoke and those who did not. Women who were interested could invite a female friend or family member to the group. The Aboriginal Research Assistants used their networks to arrange the focus groups and attendance at the NAIDOC event, reaching out by email, phone, and social media to relevant organizations (Aboriginal Community Controlled Health Services (ACCHS), women’s groups, preschools, and other community organizations). Posters advertising the groups were distributed around these community settings.

The interviewers included female Aboriginal Research Assistants (one in each location of MNC and HNE) and/or a non-Aboriginal female (student and allied health professional). The interviewers approached a potential participant prior to the focus groups or at the community event, informed them of the study, canvassed the woman’s interest in participating in the survey, and if affirmative, gained informed consent. Interviewers were trained in conducting focus groups and completing surveys. The surveys were completed on paper. On completion, women in the focus groups received a $30 shopping voucher (tobacco and alcohol purchases prohibited) for the whole session, while women recruited at the NAIDOC event and only did the survey received a $10 voucher. The study adhered to the guideline for ethical research in Aboriginal and Torres Strait Islander populations, and relevant ethics committee approvals. Approvals were obtained from the University of Newcastle Human Research Ethics Committee (reference H-2017-0247) and the Aboriginal Health and Medical Research Council Ethics Committee (AH&MRC) (reference 1303/17). This manuscript was approved by the AH&MRC on 29 July 2020.

### 2.2. Materials

The 19-item survey (Supplementary File S1) included demographic questions and questions about tobacco smoking used previously (age in years, Aboriginal and/or Torres Strait Islander identity, pregnant (Yes/No), how far into pregnancy (up to 12 weeks; 13–24 weeks; 25 weeks to term), baby or child living in your household (Yes/No), if yes, number of babies/children (1, 2, 3, 4, or more), age of children (0–12 months, 1–2 years, 3 years or older) [14]. Questions about tobacco smoking included frequency of smoking, cigarettes per day, time to the first cigarette, quit attempts, and previous use of quit medicines.

Additional questions were devised by the team with input from the Aboriginal Research Assistants and an Aboriginal senior academic. The survey included questions on the likelihood of use of community resources and social media preferences, using 5-point Likert Scales for responses (very unlikely, unlikely, neither unlikely nor likely, likely, very likely). Women were asked in response to the following 3 questions to consider each option on the Likert scale, as shown in a–h below.

1. How likely is it that in the next 3 months you will use the following supports for your health (e.g., healthy eating, exercise, mental health, and wellbeing)?
2. How likely is it that you (or a family/friend) would use the following supports to help quit smoking?
3. How likely is it that you (or a family/friend) would use the following supports for the health of a baby or small child (e.g., your own baby, niece/nephew, grandbaby)?

The questions were asked as they related to eight different categories:

- Talk to friends
- Talk to a doctor, Aboriginal Medical Service, or other health professional
- Read information on Facebook
- Talk to Elders or Aunties or other family
- Search the internet, e.g., ‘Google’
- Use a phone app (examples varied a little between questions, e.g., for health in general: ‘My QuitBuddy’, ‘Deadly Tots’, or ‘Couch to 5 km’)
- Attend a community group (examples varied between questions, e.g., sporting, healthy eating, art group, play group)
- Telephone support
- Other _____________

In relation to quitting smoking, an additional option was presented ‘Use quit medications (i.e., nicotine patches/gum etc.)’.

### 2.3. Analysis

Data collected from the paper questionnaire was entered into REDCap (Research electronic data capture) [15], hosted at Hunter Medical Research Institute, and then exported to Stata to complete the statistical analysis. All analyses were conducted using SAS Version 9.4 (SAS Institute Inc., Cary, NC, USA).

Descriptive statistics and percentages were used to describe sample characteristics. The women’s responses to the Likert scales were dichotomized into very unlikely to neither (unlikely) and very likely to likely (likely), for easier comparison. The Heaviness of Smoking Index was calculated by summing the scores from the responses to the time to first cigarette plus cigarettes per day survey questions. Descriptive characteristics such as mean, median, and frequencies were presented overall and for the subjects who responded likely/very likely to the health-related outcome questions.

Logistic regression with a Firth option was used to model the odds of respondents being likely to use the various community supports for the reasons mentioned. The Firth option addresses the issue of the small counts for some of the variables. For each outcome, individual models were built for each of the predictor variables: age, being currently pregnant, having a child/baby living in the house, and currently smoking tobacco. In the analysis, all of the models (save the age model) adjusted for age. All models utilized 98 respondents who had no data missing.

The three models/outcomes built, for example, for the first category were:

(a) How likely were you to talk to friends to help quit smoking?
(b) How likely were you to talk to friends for your health?
(c) How likely were you to talk to friends for your baby’s health?

All of the other models/categories followed this similar format.

Unbiased estimates of effect may be obtained through adjustment for potential confounders of the relationship of interest. The only potential confounder identified was age, and therefore, age is adjusted for in each model. Participants with outcomes or covariates missing were removed from the analysis so that all the models in each category had the same sample; this facilitates comparison across the category (but not between the categories). Pearson residuals were used to assess heteroscedasticity for all models.

## 3. Results

Survey data collected from the 132 eligible participants included their demographic characteristics and smoking-related behaviors and are summarized in Table 1. Age range was 16 to 79 years, with a mean of 38.79 years. Seventy-one per cent (*n* = 89) of the women were aged from 16–49 years.

Supplementary File S2 shows the descriptive analyses of the characteristics and responses of all participants, related to each type of support, and divided into whether the responses were related to quitting smoking, for their health, and the health of a baby. As an overview, these have been summarized in Table 2.

### 3.1. Regression Models

All models utilized 98 respondents, who had no data missing for the variables age, currently pregnant, baby/child in the household, and currently smoking, as well as the outcomes. Separate models were built for each predictor, and all models (except the age model) adjusted for age, see Table 3.

### 3.2. Professional Sources of Support

Health professionals: with regards to their health, odds of talking to a health professional significantly increased by 5% (1.047; 95%CI: 1.005 to 1.09; *p* = 0.0262) with each year increase in age as seen in Table 3. No other factors were significant between groups.

Telephone support: None of the investigated relationships was significantly associated with the likeliness of utilizing telephone support for any of the three purposes.

### 3.3. Community-Based Supports

Friends: women who had never smoked tobacco (vs. smokers) had 3.38 times higher odds of talking to friends about matters concerning their health. Other relationships were not statistically significant.

Family, Elder, or Auntie: with regards to talking to family about quitting smoking, their health, or the health of a baby/child, none of the modelled relationships were statistically significant.

Community groups: none of the investigated relationships were significantly associated with the likeliness of utilizing community groups for any of the three purposes.

### 3.4. Digital and Social Media Supports

#### 3.4.1. Facebook

Participants were less likely to utilize Facebook, for their health or the health of a baby/child as they got older, but there appeared to be no difference in age regarding using Facebook to help quit smoking.

#### 3.4.2. Internet

Similar to Facebook usage, older participants were less likely to use the internet for their health or that of a child/baby. They were also a lot less likely to use the internet with regards to a baby/child’s health if children were living in the household.

#### 3.4.3. Phone Apps

Women who had never smoked, compared to smokers, had two times the odds of using an app for their health, (95%CI: 1.116 to 4.187; *p* = 0.0222). Women who had quit smoking, compared to smokers, were less likely to use an app. Though not statistically significant, there was some evidence to suggest that women with a baby living in the household were also less likely to use an app for the baby/child’s health.

## 4. Discussion

In a community-based study, 132 Aboriginal and Torres Strait Islander women responded to a survey on health-seeking for themselves, a baby or child, and to quit smoking. In general, the strongest channels for health-seeking for self or a baby or child were health professionals and friends. Ninety-eight responses gave complete data that was analyzed with logistic regression models. In summary, no variables were associated with the likeliness of using telephone support, talking to family, and usage of community groups, for any of the purposes. There was an inverse linear relationship with age and the likelihood of using Facebook or the internet for women’s health or the health of a baby or child. Conversely, a positive linear relationship existed with rising age and talking to a health professional. Women who had never smoked tobacco (vs. smokers) had over three times higher odds of talking to friends about their health. When compared to smokers, women who had never smoked were more likely to utilize an app for their health, while women who had quit smoking were less likely to use an app for the same purpose. Women who had a baby or child living in their household were less likely to use the internet for a baby or child’s health, and there was weak but not statistically significant evidence to suggest they were less likely to use an app as well.

There is limited data about Aboriginal and Torres Strait Islander people accessing health services, but in general, Aboriginal and Torres Strait Islanders have significantly lower levels of usage of Medicare-rebatable services than other Australians [5,16]. In general, 89% of Australian women reported visiting a GP in the previous 12 months [13]. Younger women proportionately claim less Medicare rebates compared to older women, but Medicare data is less reliable for Aboriginal and Torres Strait Islander women accessing ACCHS, as not all services are rebatable [5,13]. Rural women, in general, have identified access to services as the most significant issue of concern [16]. A lack of female doctors, including Aboriginal and Torres Strait Islander service providers, can also deter women from seeking help [16].

Access to primary health services is vitally important for Aboriginal and Torres Strait Islander people and the issue is more than just about conveniently located services, but includes affordability, social, and cultural factors [5,12,17]. Several excellent models of care are available, such as ACCHS and for pregnant women, Aboriginal Maternal and Infant Health Services (AMIHS) antenatal services in NSW or similarly targeted services in other states [5,7]. The availability of known and trusted care providers is important in health services in general [17], and perhaps more so in pregnancy care [18]. Targeted services can provide welcoming spaces and opportunities for holistic care [17]. Where health providers work in generalized services, there may be a higher likelihood of barriers such as racism, discrimination, high costs, and communication challenges [12]. The constant presence of systemic racism, as well as interpersonal racism, can negatively influence decisions about health behaviours and health-seeking [19]. Community-driven responses have been suggested to contextualize the local barriers that require attention and thus bring about improvements [17].

The Aboriginal and Torres Strait Islander women we surveyed used a variety of types of support, including seeing health professionals. Women were likely to access health professionals (69%) for their health and 72% for the health of a baby and over 61% for quitting smoking. In another community-based survey, similarly, 63% of Aboriginal and Torres Strait Islander men and women who smoked consulted with a health professional about their smoking, and were nearly four times more likely to intend to quit smoking if they had done so, however, 40% rated the smoking cessation care they received as poor [20].

Other factors which may influence health-seeking are the cultural competence of health professionals and services, knowledge of targeted services, health literacy, supportive social networks, and the trustworthiness of information sources.

Cultural barriers lead to unequal health among Aboriginal and Torres Strait Islander people in Australia [21]. Communications between Aboriginal and non-Aboriginal people may be influenced by paternalistic interactions and distrust, which may reduce the influence of non-Aboriginal sources of information on Aboriginal and Torres Strait Islander people [19]. Cultural competence can be learned and depends on an ongoing commitment by health professionals and services alike [22]. Communication skills have been found to be inadequate among clinicians consulting pregnant women about their smoking; this may differentially affect clinicians working with Aboriginal and Torres Strait Islander women who smoke [23]. Aboriginal and Torres Strait Islander women reported receiving mixed messages and inadequate support from health providers regarding cessation [24]. General Practitioners (GPs) may be unaware of targeted or enhanced cessation services such as Aboriginal and Torres Strait Islander counsellors on the Quitline, which impairs these services in promoting telephone support [23].

On the whole, Aboriginal and Torres Strait Islander patients have good experiences of health communication with GPs [25]. Excellent interpersonal skills and avoiding medical terminology is considered best practice [25]. A more relaxed style of clinical yarning could be productive for both parties [26]. Visual aids are important for all ages and health conditions, including elderly patients, who may have lower levels of health literacy [25]. GPs also have reported a lack of suitable visual resources for smoking cessation care of pregnant Aboriginal and Torres Strait Islander women [23].

Fifty-six percent of women in this study were likely to speak with family or an Elder about their health, and 70% were likely to speak with friends, making friends one of the strongest channels for health-seeking on a par with health professionals. Strong family connections and obligations serve to provide support for the health and wellbeing of Aboriginal and Torres Strait Islander people [19]. Aboriginal and Torres Strait Islanders may be more likely to listen to the advice provided by their relatives or other Aboriginal and Torres Strait Islander people [19]. Family members often share information resources [19]. Respected individuals such as Elders serve as role models within the community and may provide examples of and support for healthy behaviours [19]. However, social networks can play an important role in perpetuating smoking behaviour [27]. Sometimes engaging in risk behaviours can lead to criticism, which may influence who Aboriginal and Torres Strait Islander people choose to seek support from [19,28]. This criticism means women who smoked may be much less likely to talk with friends about their health. Aboriginal and Torres Strait Islander women who smoke have been previously reported to have ambivalence about seeking support [24]. Women may have a reticence due to perceived stigma, a desire not to burden others, or as explained by Bovill et al. “quitting smoking was something to be undertaken on your own”.

For their health, 45% of women in this study used Facebook, 51% used the internet, and 32% used an app; whereas only 35% used Facebook, 43% used the internet, and 28% used an app for the health of a baby. Only 23% used Facebook, 37% used the internet, and 27% used an app for quitting smoking (related to either their smoking or that of a friend or family member). Aboriginal and Torres Strait Islander women are previously reported to be high users of social media such as Facebook, and community connectivity can be a key to engage women for delivering targeted solutions and improved access to health services [29]. Mobile phones are used extensively by Aboriginal and Torres Strait Islander people, including those in rural and remote areas [30]. Digital online features and social media can be used to maintain peer and family connections [29], thus supporting Indigenous priorities of community and communication. The Indigenous health sector is reported to be at the forefront of the innovative use of social media for public health promotion, among other uses, in Australia [31]. Indigenous media has a critical role in strengthening Indigenous identity and challenge stereotypes [32]. Social media has served ACCHS and other services well by enabling easy user-generation and dissemination of content [31]. However, a significant gap in the access to digital technologies in remote and regional Aboriginal and Torres Strait Islander communities needs to be addressed at a structural level to promote equity of usage [33]. The assumption that increasing age may be a barrier to the use of health information technologies has not been borne out in other populations, and it may be worthy of further exploration in this context [34].

Acceptance of health information from non-Indigenous sources or non-Indigenous perspectives may be diminished by the influence of racism and a desire of Aboriginal and Torres Strait Islander communities for cultural distinctiveness [19]. Indigenous-targeted health messages are preferred [35]. Government sources of information can be distrusted or discounted related to smoking [28]. Aboriginal and Torres Strait Islander services, including ACCHS, can provide more nuanced and culturally responsive information related to tobacco smoking, using values important to Aboriginal and Torres Strait Islander people [36]. In another study, Aboriginal and Torres Strait Islander women viewing potential printed and video resources for smoking cessation demonstrated an interest in including information sources in social media, and apps that included video content [37]. Women considered it essential to have information that was framed positively and was motivating [37]. However, women in this study were less likely to use an app if they were smoking.

In general, there is little guidance for consumers to judge the trustworthiness of information received on the internet or via mobile apps. The top 10 free maternal and child health apps were assessed by Scott et al., from Google and Apple app stores for features, including health professional input. Only four out of the ten had involved health professionals or were evidence-based [38]. There were also technical and functionality issues with the majority and only two were fully usable [38]. There has only been one trial of a targeted smoking cessation app for Aboriginal and Torres Strait Islanders, which had poor uptake and implementation challenges impacting on feasibility, which demonstrates the challenges of research in this field [39]. The uptake of the app might have been different if the app had been available on ‘general release’ through app stores [39]. The trustworthiness of apps or the lack of culturally targeted apps may reflect the lower use of apps in the women we surveyed.

### 4.1. Strengths and Limitations

The survey was conducted face-to-face by female Aboriginal Research Assistants, enabling direct interaction with the individuals, which was a strength of the study. However, respondent and social desirability bias cannot be ruled out. A further strength was that the age range of the participants was broad and included young women and Elders, both pregnant and non-pregnant women, those who smoked and those who did not, and those with and without a child at home. Only selected demographic data was collected; it did not include educational attainment, which may have limited the analysis. As the study was only conducted in two regions of NSW, the findings may not be generalizable to all Aboriginal and Torres Strait Islander women in Australia. The regression models were completed on only 98 (74%) of the respondents that had complete data. However, the statistician authors considered that this did not impact on the findings. In a field where little is known about Aboriginal and Torres Strait Islander consumer preferences for health-seeking, this research opens a window to understand how Aboriginal and Torres Strait Islander women in these regional centers are likely to access various sources of support. This initial data can give a more nuanced picture to improve accessibility and preferences.

### 4.2. Implications for Practice and Policy

Options for health information sources and assistance are rapidly expanding with the use of digital technologies. Keeping pace with these options can be a challenge. This timely study revealed that, for Aboriginal and Torres Strait Islander women in this study, the traditional avenues of health professional support are widely used. However, they are equal to the likelihood of talking to friends. Knowing that women who smoke may be more or less likely to use some of the avenues for support is relevant to organizations that are developing smoking cessation and other health promotion strategies. Women with a child in the home may be more cautious about the trustworthiness of digital resources and shy away from those that do not feel culturally safe, or they may have less time to use an app or the internet. Therefore, it is essential to involve end-users in design to ensure these aspects are accounted for when designing a targeted app or digital resources, and to understand the nuances that may promote or deter access.

There are several guiding principles in general to build the perceived trustworthiness of digital information sources. For Aboriginal and Torres Strait Islander people, the fit (surface structure) and cultural values (deep structure) are likely to both be important [40]. For anti-tobacco messages, these are already being taken account of by Aboriginal and Torres Strait Islander organizations and services developing such methods in Australia [36]. However, it is early days for developing health apps targeted for Aboriginal and Torres Strait Islander people [30]. The user experience needs to be carefully navigated.

## 5. Conclusions

In a community-based study of 132 eligible Aboriginal and Torres Strait Islander women on health-seeking for themselves, a baby or child, and to quit smoking, the strongest channels for health-seeking for self or a baby or child were health professionals and friends. A regression analysis revealed differences in the likelihood of Aboriginal and Torres Strait Islander women accessing channels of support related to age, smoking status, and having a baby or child in the home. These differences may aid in developing targeted approaches via health services, community-based, or digital and social media platforms for Indigenous women in NSW, Australia.

## Figures and Tables

**Table 1:** Demographic Characteristics of Participants.

Variable	Option	Total (*N* = 132)
Age(years)	n	126
	mean (SD)	38.79 (14.7)
	median (min, max)	36.5 (0, 79)
Dichotomized Age	16–49 years	89 (71%)
	50+ years	36 (29%)
	Missing	7
Do you identify as Aboriginal or Torres Strait Islander?	Yes, Aboriginal	124 (96%)
	Yes, Aboriginal and Torres Strait Islander	5 (3.9%)
	Missing	3
Are you currently pregnant?	No	119 (94%)
	Yes	8 (6.3%)
	Missing	5
How far into your pregnancy are you? (if pregnant)	up to 12 weeks	1 (13%)
	13 to 24 weeks	2 (25%)
	25 to 40 weeks	5 (63%)
	Missing	124
Have you given birth before? (if not pregnant)	No	20 (24%)
	Yes	65 (76%)
	Missing	47
Is a baby or a child living in your household?	yes	78 (62%)
	no	47 (38%)
	Missing	7
If yes, how many babies and/or children are living in your household?	No Children	47 (38%)
	1 child	13 (10%)
	2 children	20 (16%)
	3 children	22 (18%)
	4 or more children	23 (18%)
	Missing	7
The number of tobacco smokers in the household?	0	47 (37%)
	1	43 (34%)
	2-3	35 (27%)
	More than 3	3 (2.3%)
	Missing	4
How does your household manage places where smoking is allowed?	A complete ban on smoking anywhere inside	22 (18%)
	Smoking only on verandah/immediate area	91 (73%)
	Smoking allowed in some rooms and on the veranda	7 (5.6%)

**Table 2:** Summary of supports likely or very likely to be used for health-seeking by 132 Aboriginal women.

Type of Support	To Quit Smoking *n* (%)	Your Health *n* (%)	For Baby/Child’s Health *n* (%)
Health professional	81 (61.4%)	91 (68.9%)	96 (72.7%)
Telephone	34 (25.7%)	39 (29.5%)	44 (33.3%)
Family	64 (48.5%)	76 (57.6%)	86 (65.1%)
Friends	83 (62.9%)	92 (69.7%)	93 (70.5%)
Community Groups	46 (34.8%)	73 (55.3%)	67 (50.8%)
Facebook	30 (22.7%)	59 (44.7%)	46 (34.8%)
Internet	49 (37.1%)	67 (50.7%)	57 (43.2%)
Apps	35 (26.5%)	42 (31.8%)	37 (28%)
Other support	34 (25.7%)	39 (29.5%)	44 (33.3%)
Quit Medications	64 (48.5%)	-	-

**Table 3:** Regression models for likelihood of accessing various sources of support.

Support Type	Variable	Response	To Help Quit Smoking		For Your Own Health		For a Baby/Child’s Health	
			O.R. (95% C.I.)	*p*-Value	O.R. (95% C.I.)	*p*-Value	O.R. (95% C.I.)	*p*-Value
**Health Professionals**	Age (years)		1.026 (0.992–1.061)	0.1329	1.047 (1.005–1.09)	0.0262	1.009 (0.961–1.058)	0.7272
	Currently Pregnant (vs. not currently pregnant)		2.426 (0.338–17.415)	0.3783	6.641 (0.293–150.46)	0.2343	0.529 (0.068–4.12)	0.5429
	Baby/Child living in household (vs. no child)		1.987 (0.706–5.59)	0.1933	1.199 (0.394–3.653)	0.7494	0.555 (0.147–2.086)	0.3830
	Currently Smoke Tobacco (vs. Smokers)	No, Never smoked tobacco			1.806 (0.758–4.301)	0.1820	1.163 (0.425–3.184)	0.7682
		No, I have quit			0.715 (0.336–1.522)	0.3836	0.679 (0.278–1.658)	0.3957
**Telephone Support**	Age (years)		1.016 (0.985–1.047)	0.3207	1 (0.971–1.029)	0.9889	0.993 (0.966–1.022)	0.6452
	Currently Pregnant (vs. not currently pregnant)		0.159 (0.007–3.539)	0.2452	2.548 (0.524–12.39)	0.2465	0.919 (0.191–4.431)	0.9164
	Baby/Child living in household (vs. no child)		0.895 (0.342–2.342)	0.8217	0.947 (0.385–2.331)	0.9057	0.69 (0.286–1.661)	0.4071
	Currently Smoke Tobacco (vs. Smokers)	No, Never smoked tobacco		.	1.277 (0.686–2.377)	0.4400	1.204 (0.654–2.216)	0.5509
		No, I have quit		.	1.089 (0.585–2.028)	0.7880	1.187 (0.647–2.177)	0.5808
**Friends**	Age (years)		0.99 (0.96–1.021)	0.5223	1.01 (0.977–1.045)	0.5418	0.999 (0.962–1.038)	0.9723
	Currently Pregnant (vs. not currently pregnant)		0.395 (0.08–1.955)	0.2553	1.476 (0.205–10.634)	0.6991	0.762 (0.102–5.691)	0.7912
	Baby/Child living in household (vs. no child)		2.69 (0.904–8.01)	0.0754	1.763 (0.603–5.158)	0.3006	0.606 (0.196–1.879)	0.3860
	Currently Smoke Tobacco (vs. Smokers)	No, Never smoked tobacco		.	3.379 (1.053–10.849)	0.0407	0.876 (0.389–1.976)	0.7505
		No, I have quit		.	0.781 (0.336–1.816)	0.5658	1.107 (0.487–2.515)	0.8089
**Family**	Age (years)		1.011 (0.982–1.041)	0.4717	1.023 (0.992–1.055)	0.1552	0.991 (0.958–1.026)	0.6180
	Currently Pregnant (vs. not currently pregnant)		1.068 (0.222–5.141)	0.9347	1.668 (0.312–8.911)	0.5495	1.193 (0.164–8.703)	0.8617
	Baby/Child living in household (vs. no child)		1.723 (0.696–4.266)	0.2391	1.002 (0.402–2.498)	0.9963	0.647 (0.234–1.792)	0.4027
	Currently Smoke Tobacco (vs. Smokers)	No, Never smoked tobacco		.	1.656 (0.828–3.314)	0.1538	1.302 (0.594–2.854)	0.5103
		No, I have quit		.	1.097 (0.572–2.104)	0.7799	1.029 (0.492–2.152)	0.9388
**Community Groups**	Age (years)		1.003 (0.975–1.032)	0.8371	1.029 (0.998–1.061)	0.0681	1.007 (0.978–1.036)	0.6587
	Currently Pregnant (vs. not currently pregnant)		1.215 (0.251–5.872)	0.8085	1.774 (0.331–9.51)	0.5035	1.215 (0.251–5.872)	0.8085
	Baby/Child living in household (vs. no child)		1.053 (0.437–2.537)	0.9081	0.741 (0.299–1.838)	0.5179	0.611 (0.251–1.484)	0.2764
	Currently Smoke Tobacco (vs. Smokers)	No, Never smoked tobacco		.	1.3 (0.657–2.573)	0.4509	1.672 (0.806–3.466)	0.1674
		No, I have quit		.	1.012 (0.533–1.922)	0.9702	1.31 (0.67–2.561)	0.4295
**Facebook**	Age (years)		0.993 (0.962–1.024)	0.6426	0.945 (0.914–0.977)	0.0009	0.966 (0.937–0.996)	0.0273
	Currently Pregnant (vs. not currently pregnant)		1.094 (0.203–5.897)	0.9164	1.17 (0.214–6.391)	0.8558	0.747 (0.154–3.625)	0.7174
	Baby/Child living in household (vs. no child)		0.923 (0.351–2.428)	0.8711	0.503 (0.193–1.315)	0.1614	0.431 (0.167–1.111)	0.0814
	Currently Smoke Tobacco (vs. Smokers)	No, Never smoked tobacco		.	1.868 (0.92–3.791)	0.0837	1.407 (0.739–2.681)	0.2991
		No, I have quit		.	0.623 (0.323–1.201)	0.1580	1.112 (0.591–2.09)	0.7423
**Internet**	Age (years)		0.98 (0.952–1.009)	0.1797	0.966 (0.937–0.995)	0.0239	0.969 (0.94–0.998)	0.0369
	Currently Pregnant (vs. not currently pregnant)		0.469 (0.088–2.508)	0.3760	1.352 (0.251–7.287)	0.7257	1.534 (0.286–8.236)	0.6180
	Baby/Child living in household (vs. no child)		0.69 (0.28–1.702)	0.4205	0.489 (0.196–1.217)	0.1241	0.296 (0.116–0.754)	0.0107
	Currently Smoke Tobacco (vs. Smokers)	No, Never smoked tobacco		.	1.161 (0.6–2.247)	0.6580	1.495 (0.768–2.911)	0.2372
		No, I have quit		.	0.996 (0.527–1.883)	0.9898	0.784 (0.417–1.472)	0.4483
**Apps**	Age (years)		0.986 (0.955–1.018)	0.3957	0.983 (0.954–1.013)	0.2760	0.99 (0.96–1.02)	0.5144
	Currently Pregnant (vs. not currently pregnant)		0.12 (0.005–2.665)	0.1801	1.832 (0.379–8.851)	0.4514	1.346 (0.278–6.523)	0.7122
	Baby/Child living in household (vs. no child)		0.853 (0.323–2.248)	0.7471	0.459 (0.177–1.19)	0.1093	0.369 (0.134–1.015)	0.0534
	Currently Smoke Tobacco (vs. Smokers)	No, Never smoked tobacco		.	2.162 (1.116–4.187)	0.0222	1.28 (0.674–2.429)	0.4509
		No, I have quit		.	0.374 (0.183–0.767)	0.0073	0.858 (0.453–1.624)	0.6384

## Supplementary Materials

The following are available online at https://www.mdpi.com/1660-4601/17/21/7766/s1. File S1 shows the survey questions; File S2 shows the descriptive analyses of the characteristics and responses of all participants, related to each type of support and divided into whether the responses were related to quitting smoking, for their own health and the health of a baby.

## References

[1] Smith R., Mohapatra L., Hunter M., Evans T.-J., Oldmeadow C., Holliday E., Hure A., Attia J. (2019). A case for not adjusting birthweight customized standards for ethnicity: Observations from a unique Australian cohort. Am. J. Obset. Gynecol..

[2] Thrift A.P., Nancarrow H., Bauman A.E. (2011). Maternal smoking during pregnancy among Aboriginal women in New South Wales is linked to social gradient. Aust. N. Z. J. Public Health.

[3] Thomas D.P., Briggs V., Anderson I., Cunningham J. (2008). The social determinants of being an Indigenous non-smoker. Aust. N. Z. J. Public Health.

[4] Brady M. (2002). Historical and cultural roots of tobacco use among Aboriginal and Torres Strait Island people. Aust. N. Z. J. Public Health.

[5] Australian Institute of Health Welfare (2011). Access to Health and Services for Aboriginal and Torres Strait Islander People.

[6] Australian Indigenous HealthInfoNet Tackling Indigenous Smoking Resource and Information Centre. http://tacklingsmoking.org.au/about-the-tackling-indigenous-smoking-resource-information-centre/.

[7] Murphy E., Best E. (2012). The Aboriginal Maternal and Infant Health Service: A decade of achievement in the health of women and babies in NSW. NSW Public Health Bull..

[8] Gould G.S., Cadet-James Y., Clough A.R. (2016). Getting over the shock: Taking action on Indigenous maternal smoking. Aust. J. Prim. Health.

[9] Gould G.S., Twyman L., Stevenson L., Gribbin G.R., Bonevski B., Palazzi K., Bar-Zeev Y. (2019). What components of smoking cessation care during pregnancy are implemented by health providers: A systematic review and meta-analysis. BMJ Open.

[10] Bar-Zeev Y., Bonevski B., Twyman L., Watt K., Atkins L., Palazzi K., Oldmeadow C., Gould G.S. (2017). Opportunities missed: A Cross-Sectional Survey of the Provision of Smoking Cessation Care to Pregnant Women by Australian General Practitioners and Obstetricians. Nicotine Tob. Res..

[11] Bar-Zeev Y., Lim L.L., Bonevski B., Gruppetta M., Gould G.S. (2018). Nicotine replacement therapy for smoking cessation in pregnancy. Med. J. Aust..

[12] Davy C., Harfield S., McArthur A., Munn Z., Brown A. (2016). Access to primary health care services for Indigenous peoples: A framework synthesis. Int. J. Equity Health.

[13] Australian Institute of Health Welfare (2019). The Health of Australia’s Females.

[14] Gould G.S., Watt K., McEwen A., Cadet-James Y., Clough A.R. (2014). Validation of risk assessment scales and predictors of intentions to quit smoking in Australian Aboriginal and Torres Strait Islander peoples: A cross-sectional survey protocol. BMJ Open.

[15] Harris P.A., Taylor R., Thielke R., Payne J., Gonzalez N., Conde J.G. (2009). Research electronic data capture (REDCap)—A metadata-driven methodology and workflow process for providing translational research informatics support. J. Biomed. Inform..

[16] Australian Government Department of Health Development of a New National Women’s Health Policy Consultation Discussion Paper 2009: 5.2.3 Barriers to accessing Health Care Access. https://www1.health.gov.au/internet/publications/publishing.nsf/Content/whdp-09~whdp-09-ch5~whdp-09-ch5-2~whdp-09-ch5-2-3.

[17] Askew D., Brady J., Brown A., Cass A., Davy C., DeVries J., Fewquandie B., Hackett M., Howard M., Ingram S. (2014). To Your Door: Factors that Influence Aboriginal and Torres Strait Islander Peoples Seeking Care.

[18] Reibel T., Morrison L., Griffin D., Chapman L., Woods H. (2015). Young Aboriginal women’s voices on pregnancy care: Factors encouraging antenatal engagement. Women Birth.

[19] Waterworth P., Pescud M., Braham R., Dimmock J., Rosenberg M. (2015). Factors Influencing the Health Behaviour of Indigenous Australians: Perspectives from Support People. PLoS ONE.

[20] Gould G., Watt K., McEwen A., Cadet-James Y., Clough A. (2015). Predictors of intentions to quit smoking in Aboriginal tobacco smokers of reproductive age in regional New South Wales (NSW), Australia: Quantitative and qualitative findings of a cross-sectional survey. BMJ Open.

[21] Li J.-L. (2017). Cultural barriers lead to inequitable healthcare access for aboriginal Australians and Torres Strait Islanders. Chin. Nurs. Res..

[22] Abbott P., Dave D., Gordon E., Reath J. (2014). What do GPs need to work more effectively with Aboriginal patients? Views of Aboriginal cultural mentors and health workers. Aust. Fam. Physician.

[23] Bar-Zeev Y., Skelton E., Bonevski B., Gruppetta M., Gould G.S. (2019). Overcoming Challenges to Treating Tobacco use During Pregnancy—A Qualitative study of Australian General Practitioners Barriers. BMC Pregnancy Childbirth.

[24] Bovill M., Gruppetta M., Cadet-James Y., Clarke M., Bonevski B., Gould G.S. (2018). Wula (Voices) of Aboriginal women on barriers to accepting smoking cessation support during pregnancy: Findings from a qualitative study. Women Birth.

[25] Lakhan P., Askew D., Harris M.F., Kirk C., Hayman N. (2017). Understanding health talk in an urban Aboriginal and Torres Strait Islander primary healthcare service: A cross-sectional study. Aust. J. Prim. Health.

[26] Lin I., Green C., Bessarab D. (2016). ‘Yarn with me’: Applying clinical yarning to improve clinician-patient communication in Aboriginal health care. Aust. J. Prim. Health.

[27] Passey M., Gale J., Sanson-Fisher R. (2011). “It’s almost expected”: Rural Australian Aboriginal women’s reflections on smoking initiation and maintenance: A qualitative study. BMC Womens Health.

[28] Gould G.S., Munn J., Avuri S., Hoff S., Cadet-James Y., McEwen A., Clough A.R. (2013). “Nobody smokes in the house if there’s a new baby in it”: Aboriginal perspectives on tobacco smoking in pregnancy and in the household in regional NSW Australia. Women Birth.

[29] Rice E.S., Haynes E., Royce P., Thompson S.C. (2016). Social media and digital technology use among Indigenous young people in Australia: A literature review. Int. J. Equity Health.

[30] Brusse C., Gardner K., McAullay D., Dowden M. (2014). Social Media and Mobile Apps for Health Promotion in Australian Indigenous Populations: Scoping Review. J. Med. Internet. Res..

[31] Sweet M.A. (2013). Social media: New links for Indigenous health. Med. J. Aust..

[32] Australian Human Rights Commission Indigenous Media Strengthens Identity. http://humanrights.gov.au/about/media/media_releases/2012/61_12.html.

[33] Smith K., Chenhall R., McGuire S., Kowal E. (2016). Digital Futures in Indigenous Communities, Melbourne Networked Society Institute Research Paper, No. 3.

[34] Miller N.A., Kirk A., Alston B., Glos L. (2013). Effects of Gender, Disability, and Age in the Receipt of Preventive Services. Gerontologist.

[35] Gould G.S., McEwen A., Watters T., Clough A.R., van der Zwan R. (2013). Should anti-tobacco media messages be culturally targeted for Indigenous populations? A systematic review and narrative synthesis. Tob. Control.

[36] Gould G.S., Watt K., Stevenson L., McEwen A., Cadet-James Y., Clough A.R. (2014). Developing anti-tobacco messages for Australian Aboriginal and Torres Strait Islander peoples: Evidence from a national cross-sectional survey. BMC Public Health.

[37] Bovill M., Bar-Zeev Y., Gruppetta M., Clarke M., Nicholls K., O’Mara P., Bonevski B., Reath J., Gould G. (2018). Giri-nya-la-nha (talk together) to explore acceptability of targeted smoking cessation resources with Australian Aboriginal women. Public Health.

[38] Scott K., Gome G., Richards D., Caldwell P. (2015). Trustworthiness of apps for maternal and child health: Assessing technical performance and evidence based content. J. Paediatr. Child Health.

[39] Peiris D., Wright L., News M., Rogers K., Redfern J., Chow C., Thomas D. (2019). A Smartphone App to Assist Smoking Cessation Among Aboriginal Australians: Findings From a Pilot Randomized Controlled Trial. JMIR mHealth uHealth.

[40] Resnicow K., Baranowski T., Ahluwalia J.S., Braithwaite R.L. (1999). Cultural sensitivity in public health: Defined and demystified. Ethn. Dis..

