# Supplementary material for: Supports Used by Aboriginal and Torres Strait Islander Women for Their Health, including Smoking Cessation, and a Baby’s Health: A Cross-Sectional Survey in New South Wales, Australia
Source: Int J Environ Res Public Health. 2020 Oct 23;17(21):7766. doi: 10.3390/ijerph17217766 (PMC7660307; doi:10.3390/ijerph17217766)
Supplement: Supplementary file 1 [file ijerph-17-07766-s001.pdf]

Supplementary File 1: Survey Questions

Section 1: First some questions about yourself and your household

1. What is your age ______________

2. Do you identify as Aboriginal or Torres Strait Islander?

- ☐ Yes, Aboriginal
- ☐ Yes, Torres Strait Islander
- ☐ Yes, Aboriginal & Torres Strait Islander
- ☐ Not, Aboriginal & Torres Strait Islander [exclude from study]

3. Are you currently pregnant?

- ☐ Yes
- ☐ No

A. If yes: how far into your pregnancy are you?

- ☐ Up to 12 weeks
- ☐ 13-24 weeks
- ☐ 25 weeks to 40 weeks (to term)

B. If no: have you given birth before?

- ☐ Yes
- ☐ No

4. Is a baby or child living in your household?

- ☐ Yes
- ☐ No

A. If yes, how many babies and/or children are living in your household?

- ☐ 1 child
- ☐ 2 children
- ☐ 3 children
- ☐ 4 or more children

B. How old are the children (tick any boxes that apply)

- ☐ 0 to 12 months
- ☐ 1 to 2 years
- ☐ 3 years and older

Section 2: These questions are about tobacco smoking

5. How many tobacco smokers (include yourself if you still smoke) usually live in your household?

- ☐ 0
- ☐ 1
- ☐ 2–3
- ☐ More than 3

6. How does your household manage places where smoking is allowed?

- ☐ Complete ban on smoking anywhere inside the house or outside
- ☐ Smoking only on verandah/immediate area outside (not inside)
- ☐ Smoking allowed in some rooms and on verandah/immediate area outside
- ☐ People can smoke anywhere in or outside

7. Do you currently smoke tobacco?

- ☐ **Yes**
- ☐ **No** I used to smoke tobacco but have quit completely (Skip to section 3)
- ☐ **No** I have never smoked tobacco of any type (Skip to section 3)

For those who are currently smoking only:

8. How often do you smoke tobacco?

- ☐ Every day
- ☐ Most days (4 or more days a week, but not every day)
- ☐ Some days (1–3 days a week)
- ☐ Very occasionally (less than once a week)

9. How many cigarettes do you smoke per day when you do smoke?

- ☐ 10 or less
- ☐ 11–20
- ☐ 21–30
- ☐ 31 or more

10. How soon do you have your first smoke after waking in the morning when you do smoke?

- ☐ Within 5 minutes
- ☐ 6–30 minutes
- ☐ 31–60 minutes
- ☐ Over 60 minutes

11. Have you ever tried to quit smoking?

- ☐ Yes I am trying to quit now
- ☐ Yes, in the last 3 months
- ☐ Yes, between 6 – 12 months ago
- ☐ Yes, over one year ago
- ☐ No, I have never tried to quit smoking

12. **What have you used to quit smoking?**

- ☐ Nicotine patches
- ☐ Nicotine gum
- ☐ I quit cold turkey
- ☐ Other,_____________________
- ☐ I haven’t tried to quit smoking

Section 3: these questions are about community supports you use for your health

Please answer each question by choosing which response applies to you depending on how likely you are take the following actions

**13.** How likely is it that you (or a family/friend) would use the following supports to help quit smoking:	*Very unlikely*	*Unlikely*	*Neither likely or unlikely*	*Likely*	*Very likely*
a. Talk to friends	1	2	3	4	5
b. Talk to a Doctor, Aboriginal Medical Service or other health professional	1	2	3	4	5
c. Use quit medications (i.e. Nicotine patches/gum etc.)	1	2	3	4	5
d. Get support on Facebook i.e. follow a page or post about your quitting	1	2	3	4	5
e. Talk to elders or Aunties or other family	1	2	3	4	5
f. Search the internet e.g. 'Google' for advice and information	1	2	3	4	5
g Use a phone App e.g. 'My QuitBuddy'	1	2	3	4	5
h. Attend a community group e.g. health group	1	2	3	4	5
i, Telephone support	1	2	3	4	5
j. other _________________	1	2	3	4	5

Please answer <u>each question</u> by choosing which response applies to you depending on how likely you are take the following actions

**14.** **How likely is it that in the next 3 months you will use the following supports for your health (e.g. healthy eating, exercise, mental health and wellbeing):**	***Very unlikely***	***Unlikely***	***Neither likely or unlikely***	***Likely***	***Very likely***
**a. Talk to friends**	**1**	**2**	**3**	**4**	**5**
**b. Talk to a Doctor, Aboriginal Medical Service or other health professional**	**1**	**2**	**3**	**4**	**5**
**c. Read information on Facebook**	**1**	**2**	**3**	**4**	**5**
**d. Talk to elders or Aunties or other family**	**1**	**2**	**3**	**4**	**5**
**e. Search the internet e.g. ‘Google’**	**1**	**2**	**3**	**4**	**5**
**f. Use a phone App e.g. ‘My QuitBuddy’, ‘Deadly Tots’ , ‘Couch to 5km’,**	**1**	**2**	**3**	**4**	**5**
**g. Attend a community group e.g. sporting, healthy eating, art group**	**1**	**2**	**3**	**4**	**5**
**h. Use telephone support**	**1**	**2**	**3**	**4**	**5**
**i. Other _________________**	**1**	**2**	**3**	**4**	**5**

Please answer <u>each question</u> by choosing which response applies to you depending on how likely you are take the following actions

15. **How likely is it that you (or a family/friend) would use the following supports for the health of a baby or small child (e.g. your own baby, niece/nephew, grandbaby):**	***Very unlikely***	***Unlikely***	***Neither likely or unlikely***	***Likely***	***Very likely***
**a. Talk to friends**	**1**	**2**	**3**	**4**	**5**
**b. Talk to a Doctor, Aboriginal Medical Service or other health professional**	**1**	**2**	**3**	**4**	**5**
**c. Use Facebook or other social media site for support or advise**	**1**	**2**	**3**	**4**	**5**
**d. Talk to elders or Aunties or other family**	**1**	**2**	**3**	**4**	**5**
**e. Search the internet e.g. 'Google'**	**1**	**2**	**3**	**4**	**5**
**f. Use a phone App e.g. 'Deadly Tots'**	**1**	**2**	**3**	**4**	**5**
**g. Attend a community group e.g. playgroup**	**1**	**2**	**3**	**4**	**5**
**h. Telephone support**	**1**	**2**	**3**	**4**	**5**
**h. other ________________**	**1**	**2**	**3**	**4**	**5**

Supplementary Table 1: ***Characteristics of participants that talk to a health professional***

Question	Option	To Quit Smoking (n=81)	Your Health (n=91)	For a Baby (n=96)
What is your age?	n	80	89	94
	mean (SD)	39.23 (14.18)	40.8 (14.43)	39.16 (14.32)
	median (min, max)	38.5 (0, 67)	39 (0, 79)	37.5 (0, 68)
Dichotomised Age	16-49 years	55 (70%)	60 (68%)	66 (71%)
	50+ years	24 (30%)	28 (32%)	27 (29%)
	Missing	2	3	3
Do you identify as Aboriginal or Torres Strait Islander?	Yes, Aboriginal	77 (96%)	85 (94%)	91 (96%)
	Yes, Aboriginal & Torres Strait Islander	3 (3.8%)	5 (5.6%)	4 (4.2%)
	Missing	1	1	1
Are you currently pregnant?	No	75 (93%)	82 (92%)	88 (94%)
	Yes	6 (7.4%)	7 (7.9%)	6 (6.4%)
	Missing	0	2	2
How far into your pregnancy are you?(if pregnant)	up to 12 weeks			
	13 to 24 weeks	2 (33%)	2 (29%)	1 (17%)
	25 to 40 weeks	4 (67%)	5 (71%)	5 (83%)
	Missing	75	84	90
Have you given birth before?(if not pregnant)	No	10 (19%)	11 (19%)	11 (17%)
	Yes	44 (81%)	47 (81%)	52 (83%)
	Missing	27	33	33
If yes, how many babies and/or children are living in your household?	No Children	31 (39%)	33 (38%)	33 (35%)
	1 child	5 (6.3%)	7 (8.1%)	12 (13%)
	2 children	17 (22%)	13 (15%)	14 (15%)
	3 children	14 (18%)	16 (19%)	17 (18%)
	4 or more children	12 (15%)	17 (20%)	17 (18%)
	Missing	2	5	3
Number of tobacco smokers in household?	0	31 (39%)	39 (43%)	35 (36%)
	1	23 (29%)	25 (28%)	31 (32%)
	2-3	24 (30%)	24 (27%)	27 (28%)
	More than 3	2 (2.5%)	2 (2.2%)	3 (3.1%)
	Missing	1	1	0
How does your household manage places where smoking is allowed?	Complete ban on smoking anywhere inside	13 (16%)	19 (22%)	17 (18%)

	Smoking only on verandah/immediate area	58 (73%)	59 (68%)	66 (72%)
	Smoking allowed in some rooms & on veranda	6 (7.5%)	6 (6.9%)	7 (7.6%)
	People can smoke anywhere in or outside	3 (3.8%)	3 (3.4%)	2 (2.2%)
	Missing	1	4	4
Do you currently smoke tobacco?	Yes	35 (44%)	33 (37%)	39 (41%)
	No, I used to smoke tobacco but have quit	24 (30%)	29 (33%)	31 (33%)
	No, I have never smoked tobacco of any time	20 (25%)	27 (30%)	25 (26%)
	Missing	2	2	1
How often do you smoke tobacco?(smokers only)	Daily	26 (74%)	24 (77%)	27 (73%)
	Non-Daily	9 (26%)	7 (23%)	10 (27%)
	Missing	46	60	59
How many cigarettes do you smoke per day? (continuous variable- smokers only)	n	14	10	9
	mean (SD)	9.64 (5.02)	10.6 (4.86)	10.56 (5.29)
	median (min, max)	10 (4, 20)	10 (5, 20)	10 (4, 20)
How many cigarettes do you smoke per day? (categorial variable- smokers only)	10 or less	23 (66%)	22 (69%)	22 (58%)
	11-20	12 (34%)	10 (31%)	16 (42%)
	Missing	46	59	58
How soon do you have your first smoke?	don't smoke at all	1 (2.9%)		
	Within 5 minutes	5 (15%)	5 (17%)	5 (14%)
	6-30 minutes	18 (53%)	15 (50%)	18 (50%)
	31-60 minutes	5 (15%)	6 (20%)	6 (17%)
	Over 60 minutes	5 (15%)	4 (13%)	7 (19%)
	Missing	47	61	60
Heaviness of Smoking Index	n	34	30	36
	mean (SD)	5.12 (1.25)	5.03 (1.22)	5.03 (1.28)
	median (min, max)	5 (3, 7)	5 (3, 7)	5 (3, 7)
Have you ever tried to quit smoking? (Yes/No. Smokers only)	No, I haven't tried to quit	5 (14%)	5 (16%)	4 (11%)
	Yes, I've tried to quit	30 (86%)	27 (84%)	34 (89%)
	Missing	46	59	58

Supplementary Table 2: *Characteristics of participants that use a telephone*

Question	Option	To Quit Smoking (n=34)	Your Health (n=39)	For a Baby (n=44)
What is your age?	n	34	38	43
	mean (SD)	39.88 (13.54)	38.16 (12.35)	37.98 (13.71)
	median (min, max)	40.5 (16, 67)	37 (16, 63)	37 (0, 64)
Dichotomised Age	16-49 years	24 (71%)	29 (76%)	31 (74%)
	50+ years	10 (29%)	9 (24%)	11 (26%)
	Missing	0	1	2
Do you identify as Aboriginal or Torres Strait Islander?	Yes, Aboriginal	32 (94%)	37 (95%)	40 (93%)
	Yes, Aboriginal & Torres Strait Islander	2 (5.9%)	2 (5.1%)	3 (7.0%)
	Missing	0	0	1
Are you currently pregnant?	No	34 (100%)	35 (90%)	41 (93%)
	Yes		4 (10%)	3 (6.8%)
	Missing	0	0	0
How far into your pregnancy are you?(if pregnant)	up to 12 weeks			
	13 to 24 weeks		1 (25%)	
	25 to 40 weeks		3 (75%)	3 (100%)
	Missing	34	35	41
Have you given birth before?(if not pregnant)	No	5 (18%)	5 (19%)	4 (14%)
	Yes	23 (82%)	22 (81%)	25 (86%)
	Missing	6	12	15
If yes, how many babies and/or children are living in your household?	No Children	11 (32%)	14 (36%)	14 (32%)
	1 child	2 (5.9%)	2 (5.1%)	5 (11%)
	2 children	10 (29%)	8 (21%)	8 (18%)
	3 children	6 (18%)	6 (15%)	6 (14%)
	4 or more children	5 (15%)	9 (23%)	11 (25%)
	Missing	0	0	0
Number of tobacco Smokers in household?	0	15 (44%)	18 (46%)	18 (41%)
	1	12 (35%)	9 (23%)	12 (27%)
	2-3	7 (21%)	11 (28%)	13 (30%)
	More than 3		1 (2.6%)	1 (2.3%)
	Missing	0	0	0
How does your household manage places where smoking is allowed?	Complete ban on smoking anywhere inside	7 (21%)	11 (28%)	11 (25%)

	Smoking only on verandah/immediate area	24 (71%)	24 (62%)	30 (68%)
	Smoking allowed in some rooms & on veranda	1 (2.9%)	2 (5.1%)	3 (6.8%)
	People can smoke anywhere in or outside	2 (5.9%)	2 (5.1%)	
	Missing	0	0	0
Do you currently smoke tobacco?	Yes	13 (39%)	13 (34%)	16 (36%)
	No, I used to smoke tobacco but have quit	8 (24%)	12 (32%)	14 (32%)
	No, I have never smoked tobacco of any time	12 (36%)	13 (34%)	14 (32%)
	Missing	1	1	0
How often do you smoke tobacco?(smokers only)	Daily	12 (92%)	11 (85%)	13 (81%)
	Non-Daily	1 (7.7%)	2 (15%)	3 (19%)
	Missing	21	26	28
How many cigarettes do you smoke per day?(continuous variable-smokers only)	n	6	4	5
	mean (SD)	11.67 (4.08)	12.5 (5)	11.2 (5.22)
	median (min, max)	10 (10, 20)	10 (10, 20)	10 (6, 20)
How many cigarettes do you smoke per day?(categorial variable-smokers only)	10 or less	8 (62%)	8 (62%)	10 (63%)
	11-20	5 (38%)	5 (38%)	6 (38%)
	Missing	21	26	28
How soon do you have your first smoke?	don’t smoke at all			
	Within 5 minutes	3 (25%)	3 (25%)	2 (13%)
	6-30 minutes	6 (50%)	4 (33%)	6 (40%)
	31-60 minutes	3 (25%)	4 (33%)	4 (27%)
	Over 60 minutes		1 (8.3%)	3 (20%)
	Missing	22	27	29
Heaviness of Smoking Index	n	12	12	15
	mean (SD)	5.42 (1)	5.17 (1.34)	4.87 (1.25)
	median (min, max)	5 (4, 7)	5 (3, 7)	5 (3, 7)
Have you ever tried to quit smoking?(Yes/No. Smokers only)	No, I haven't tried to quit	1 (7.7%)	1 (7.7%)	2 (13%)
	Yes, I've tried to quit	12 (92%)	12 (92%)	14 (88%)
	Missing	21	26	28

Supplementary Table 3: ***Characteristics of participants that talk to family***

Question	Option	To Quit Smoking (n=64)	Your Health (n=76)	For a Baby (n=86)
What is your age?	n	63	74	84
	mean (SD)	38.4 (14.53)	39.61 (14.57)	39.14 (14.76)
	median (min, max)	38 (0, 68)	37.5 (0, 79)	37 (0, 79)
Dichotomised Age	16-49 years	44 (71%)	52 (71%)	59 (71%)
	50+ years	18 (29%)	21 (29%)	24 (29%)
	Missing	2	3	3
Do you identify as Aboriginal or Torres Strait Islander?	Yes, Aboriginal	60 (95%)	71 (95%)	81 (95%)
	Yes, Aboriginal & Torres Strait Islander	3 (4.8%)	4 (5.3%)	4 (4.7%)
	Missing	1	1	1
Are you currently pregnant?	No	60 (94%)	69 (93%)	78 (93%)
	Yes	4 (6.3%)	5 (6.8%)	6 (7.1%)
	Missing	0	2	2
How far into your pregnancy are you? (if pregnant)	up to 12 weeks			
	13 to 24 weeks	1 (25%)	1 (20%)	1 (17%)
	25 to 40 weeks	3 (75%)	4 (80%)	5 (83%)
	Missing	60	71	80
Have you given birth before? (if not pregnant)	No	6 (15%)	8 (16%)	10 (18%)
	Yes	33 (85%)	41 (84%)	45 (82%)
	Missing	25	27	31
If yes, how many babies and/or children are living in your household?	No Children	24 (39%)	26 (37%)	28 (34%)
	1 child	5 (8.1%)	5 (7.0%)	10 (12%)
	2 children	14 (23%)	13 (18%)	14 (17%)
	3 children	9 (15%)	12 (17%)	14 (17%)
	4 or more children	10 (16%)	15 (21%)	17 (20%)
	Missing	2	5	3
Number of tobacco Smokers in household?	0	28 (44%)	36 (48%)	33 (38%)
	1	19 (30%)	21 (28%)	26 (30%)
	2-3	15 (23%)	15 (20%)	24 (28%)
	More than 3	2 (3.1%)	3 (4.0%)	3 (3.5%)
	Missing	0	1	0
How does your household manage places where smoking is allowed?	Complete ban on smoking anywhere inside	12 (20%)	16 (22%)	17 (21%)
	Smoking only on verandah/immediate area	44 (72%)	49 (68%)	56 (68%)

	Smoking allowed in some rooms & on veranda	4 (6.6%)	5 (6.9%)	6 (7.3%)
	People can smoke anywhere in or outside	1 (1.6%)	2 (2.8%)	3 (3.7%)
	Missing	3	4	4
Do you currently smoke tobacco?	Yes	22 (35%)	24 (32%)	33 (39%)
	No, I used to smoke tobacco but have quit	21 (33%)	26 (35%)	29 (34%)
	No, I have never smoked tobacco of any time	20 (32%)	24 (32%)	23 (27%)
	Missing	1	2	1
How often do you smoke tobacco?(smokers only)	Daily	15 (71%)	16 (70%)	22 (69%)
	Non-Daily	6 (29%)	7 (30%)	10 (31%)
	Missing	43	53	54
How many cigarettes do you smoke per day? (continuous variable- smokers only)	n	8	7	9
	mean (SD)	8.75 (3.58)	9.29 (5.38)	10 (5.02)
	median (min, max)	10 (4, 15)	10 (4, 20)	10 (4, 20)
How many cigarettes do you smoke per day? (categorial variable- smokers only)	10 or less	14 (64%)	15 (63%)	20 (61%)
	11-20	8 (36%)	9 (38%)	13 (39%)
	Missing	42	52	53
How soon do you have your first smoke?	Don’t smoke at all	1 (5.0%)		
	Within 5 minutes	3 (15%)	4 (18%)	4 (13%)
	6-30 minutes	9 (45%)	9 (41%)	14 (45%)
	31-60 minutes	4 (20%)	4 (18%)	6 (19%)
	Over 60 minutes	3 (15%)	5 (23%)	7 (23%)
	Missing	44	54	55
Heaviness of Smoking Index	n	20	22	31
	mean (SD)	5.15 (1.31)	4.95 (1.4)	4.9 (1.33)
	median (min, max)	5 (3, 7)	5 (3, 7)	5 (3, 7)
Have you ever tried to quit smoking? (Yes/No. Smokers only)	No, I haven't tried to quit	3 (14%)	2 (8.3%)	2 (6.1%)
	Yes, I've tried to quit	19 (86%)	22 (92%)	31 (94%)
	Missing	42	52	53

Supplementary Table 4: ***Characteristics of participants that talk to friends***

Question	Option	To Quit Smoking (n=83)	Your Health (n=92)	For a Baby (n=93)
What is your age?	n	82	90	92
	mean (SD)	37.94 (14.19)	38.8 (14.85)	39.39 (14.45)
	median (min, max)	36.5 (0, 68)	36.5 (0, 79)	37 (0, 79)
Dichotomised Age	16-49 years	59 (73%)	64 (72%)	65 (71%)
	50+ years	22 (27%)	25 (28%)	26 (29%)
	Missing	2	3	2
Do you identify as Aboriginal or Torres Strait Islander?	Yes, Aboriginal	80 (98%)	86 (95%)	87 (95%)
	Yes, Aboriginal & Torres Strait Islander	2 (2.4%)	5 (5.5%)	5 (5.4%)
	Missing	1	1	1
Are you currently pregnant?	No	78 (95%)	84 (93%)	86 (93%)
	Yes	4 (4.9%)	6 (6.7%)	6 (6.5%)
	Missing	1	2	1
How far into your pregnancy are you?(if pregnant)	up to 12 weeks			
	13 to 24 weeks	1 (25%)	1 (17%)	1 (17%)
	25 to 40 weeks	3 (75%)	5 (83%)	5 (83%)
	Missing	79	86	87
Have you given birth before?(if not pregnant)	No	13 (24%)	13 (22%)	8 (14%)
	Yes	42 (76%)	47 (78%)	51 (86%)
	Missing	28	32	34
If yes, how many babies and/or children are living in your household?	No Children	33 (42%)	33 (38%)	31 (34%)
	1 child	8 (10%)	6 (6.9%)	11 (12%)
	2 children	14 (18%)	15 (17%)	16 (18%)
	3 children	11 (14%)	14 (16%)	15 (16%)
	4 or more children	13 (16%)	19 (22%)	18 (20%)
	Missing	4	5	2
Number of tobacco Smokers in household?	0	33 (41%)	40 (44%)	33 (35%)
	1	26 (32%)	30 (33%)	34 (37%)
	2-3	20 (25%)	18 (20%)	23 (25%)
	More than 3	2 (2.5%)	3 (3.3%)	3 (3.2%)
	Missing	2	1	0
How does your household manage places where smoking is allowed?	Complete ban on smoking anywhere inside	11 (14%)	20 (23%)	17 (19%)

	Smoking only on verandah/immediate area	60 (77%)	62 (70%)	62 (70%)
	Smoking allowed in some rooms & on veranda	5 (6.4%)	4 (4.5%)	7 (7.9%)
	People can smoke anywhere in or outside	2 (2.6%)	2 (2.3%)	3 (3.4%)
	Missing	5	4	4
Do you currently smoke tobacco?	Yes	34 (42%)	30 (33%)	39 (42%)
	No, I used to smoke tobacco but have quit	22 (27%)	30 (33%)	31 (33%)
	No, I have never smoked tobacco of any time	25 (31%)	30 (33%)	23 (25%)
	Missing	2	2	0
How often do you smoke tobacco?(smokers only)	Daily	24 (73%)	19 (68%)	28 (76%)
	Non-Daily	9 (27%)	9 (32%)	9 (24%)
	Missing	50	64	56
How many cigarettes do you smoke per day? (continuous variable- smokers only)	n	12	11	10
	mean (SD)	9.5 (5.09)	9.82 (5.21)	10.5 (4.99)
	median (min, max)	10 (4, 20)	10 (4, 20)	10 (4, 20)
How many cigarettes do you smoke per day? (categorial variable- smokers only)	10 or less	23 (68%)	18 (62%)	22 (58%)
	11-20	11 (32%)	11 (38%)	16 (42%)
	Missing	49	63	55
How soon do you have your first smoke?	don’t smoke at all	1 (3.1%)		
	Within 5 minutes	5 (16%)	6 (22%)	7 (19%)
	6-30 minutes	14 (44%)	11 (41%)	16 (44%)
	31-60 minutes	6 (19%)	4 (15%)	6 (17%)
	Over 60 minutes	6 (19%)	6 (22%)	7 (19%)
	Missing	51	65	57
Heaviness of Smoking Index	n	32	27	36
	mean (SD)	5 (1.34)	5.04 (1.4)	5.08 (1.32)
	median (min, max)	5 (3, 7)	5 (3, 7)	5 (3, 7)
Have you ever tried to quit smoking?(Yes/No. Smokers only)	No, I haven't tried to quit	5 (15%)	5 (17%)	4 (11%)
	Yes, I've tried to quit	29 (85%)	24 (83%)	34 (89%)
	Missing	49	63	55

Supplementary Table 5: ***Characteristics of participants that use community groups***

Question	Option	To Quit Smoking (n=46)	Your Health (n=73)	For a Baby (n=67)
What is your age?	n	46	71	66
	mean (SD)	38.65 (14.01)	41.07 (14.39)	39.38 (14.63)
	median (min, max)	36 (16, 68)	39 (0, 79)	37.5 (0, 79)
Dichotomised Age	16-49 years	34 (74%)	48 (69%)	47 (72%)
	50+ years	12 (26%)	22 (31%)	18 (28%)
	Missing	0	3	2
Do you identify as Aboriginal or Torres Strait Islander?	Yes, Aboriginal	44 (98%)	69 (96%)	61 (92%)
	Yes, Aboriginal & Torres Strait Islander	1 (2.2%)	3 (4.2%)	5 (7.6%)
	Missing	1	1	1
Are you currently pregnant?	No	43 (93%)	67 (93%)	61 (91%)
	Yes	3 (6.5%)	5 (6.9%)	6 (9.0%)
	Missing	0	1	0
How far into your pregnancy are you?(if pregnant)	up to 12 weeks			
	13 to 24 weeks	1 (33%)	1 (20%)	1 (17%)
	25 to 40 weeks	2 (67%)	4 (80%)	5 (83%)
	Missing	43	68	61
Have you given birth before?(if not pregnant)	No	6 (19%)	6 (13%)	6 (15%)
	Yes	25 (81%)	39 (87%)	35 (85%)
	Missing	15	28	26
If yes, how many babies and/or children are living in your household?	No Children	15 (34%)	25 (36%)	22 (33%)
	1 child	3 (6.8%)	4 (5.7%)	6 (9.1%)
	2 children	10 (23%)	12 (17%)	12 (18%)
	3 children	8 (18%)	13 (19%)	12 (18%)
	4 or more children	8 (18%)	16 (23%)	14 (21%)
	Missing	2	3	1
Number of tobacco Smokers in household?	0	21 (46%)	34 (47%)	28 (42%)
	1	13 (28%)	21 (29%)	21 (31%)
	2-3	12 (26%)	15 (21%)	16 (24%)
	More than 3		3 (4.1%)	2 (3.0%)
	Missing	0	0	0
How does your household manage places where smoking is allowed?	Complete ban on smoking anywhere inside	9 (20%)	16 (23%)	14 (22%)

	Smoking only on verandah/immediate area	32 (73%)	48 (69%)	46 (71%)
	Smoking allowed in some rooms & on veranda	2 (4.5%)	4 (5.7%)	4 (6.2%)
	People can smoke anywhere in or outside	1 (2.3%)	2 (2.9%)	1 (1.5%)
	Missing	2	3	2
Do you currently smoke tobacco?	Yes	15 (33%)	23 (32%)	20 (30%)
	No, I used to smoke tobacco but have quit	15 (33%)	26 (36%)	25 (38%)
	No, I have never smoked tobacco of any time	15 (33%)	23 (32%)	21 (32%)
	Missing	1	1	1
How often do you smoke tobacco?(smokers only)	Daily	9 (64%)	16 (73%)	14 (70%)
	Non-Daily	5 (36%)	6 (27%)	6 (30%)
	Missing	32	51	47
How many cigarettes do you smoke per day?(continuous variable-smokers only)	n	5	4	6
	mean (SD)	9.8 (3.9)	11.25 (2.5)	10.17 (5.31)
	median (min, max)	10 (4, 15)	10 (10, 15)	10 (5, 20)
How many cigarettes do you smoke per day?(categorial variable-smokers only)	10 or less	10 (67%)	13 (57%)	12 (60%)
	11-20	5 (33%)	10 (43%)	8 (40%)
	Missing	31	50	47
How soon do you have your first smoke?	don't smoke at all	1 (7.7%)		
	Within 5 minutes	3 (23%)	4 (19%)	3 (16%)
	6-30 minutes	4 (31%)	10 (48%)	10 (53%)
	31-60 minutes	5 (38%)	5 (24%)	3 (16%)
	Over 60 minutes		2 (9.5%)	3 (16%)
	Missing	33	52	48
Heaviness of Smoking Index	n	13	21	19
	mean (SD)	5.38 (1.19)	5.24 (1.14)	5.11 (1.29)
	median (min, max)	5 (4, 7)	5 (3, 7)	5 (3, 7)
Have you ever tried to quit smoking?(Yes/No. Smokers only)	No, I haven't tried to quit	3 (20%)	4 (17%)	4 (20%)
	Yes, I've tried to quit	12 (80%)	19 (83%)	16 (80%)
	Missing	31	50	47

Supplementary Table 6: ***Characteristics of participants that use Facebook***

Question	Option	To Quit Smoking (n=30)	Your Health (n=59)	For a Baby (n=46)
What is your age?	n	30	58	45
	mean (SD)	38 (13.44)	33.84 (11.75)	36.2 (13.41)
	median (min, max)	36.5 (17, 60)	31.5 (0, 61)	34 (0, 64)
Dichotomised Age	16-49 years	21 (70%)	49 (86%)	35 (80%)
	50+ years	9 (30%)	8 (14%)	9 (20%)
	Missing	0	2	2
Do you identify as Aboriginal or Torres Strait Islander?	Yes, Aboriginal	27 (90%)	54 (92%)	43 (93%)
	Yes, Aboriginal & Torres Strait Islander	3 (10%)	5 (8.5%)	3 (6.5%)
	Missing	0	0	0
Are you currently pregnant?	No	28 (93%)	54 (92%)	42 (93%)
	Yes	2 (6.7%)	5 (8.5%)	3 (6.7%)
	Missing	0	0	1
How far into your pregnancy are you?(if pregnant)	up to 12 weeks			
	13 to 24 weeks		1 (20%)	
	25 to 40 weeks	2 (100%)	4 (80%)	3 (100%)
	Missing	28	54	43
Have you given birth before?(if not pregnant)	No	4 (19%)	11 (27%)	5 (18%)
	Yes	17 (81%)	30 (73%)	23 (82%)
	Missing	9	18	18
If yes, how many babies and/or children are living in your household?	No Children	10 (33%)	17 (29%)	11 (25%)
	1 child	3 (10%)	6 (10%)	6 (14%)
	2 children	8 (27%)	9 (15%)	9 (20%)
	3 children	3 (10%)	14 (24%)	8 (18%)
	4 or more children	6 (20%)	13 (22%)	10 (23%)
	Missing	0	0	2
Number of tobacco Smokers in household?	0	15 (50%)	26 (44%)	21 (46%)
	1	10 (33%)	18 (31%)	11 (24%)
	2-3	4 (13%)	14 (24%)	13 (28%)
	More than 3	1 (3.3%)	1 (1.7%)	1 (2.2%)
	Missing	0	0	0
How does your household manage places where smoking is allowed?	Complete ban on smoking anywhere inside	8 (27%)	12 (21%)	13 (30%)

	Smoking only on verandah/immediate area	18 (60%)	42 (74%)	29 (66%)
	Smoking allowed in some rooms & on veranda	2 (6.7%)	2 (3.5%)	1 (2.3%)
	People can smoke anywhere in or outside	2 (6.7%)	1 (1.8%)	1 (2.3%)
	Missing	0	2	2
Do you currently smoke tobacco?	Yes	11 (37%)	23 (39%)	16 (35%)
	No, I used to smoke tobacco but have quit	8 (27%)	14 (24%)	16 (35%)
	No, I have never smoked tobacco of any time	11 (37%)	22 (37%)	14 (30%)
	Missing	0	0	0
How often do you smoke tobacco?(smokers only)	Daily	8 (73%)	13 (62%)	8 (53%)
	Non-Daily	3 (27%)	8 (38%)	7 (47%)
	Missing	19	38	31
How many cigarettes do you smoke per day? (continuous variable-smokers only)	n	4	7	5
	mean (SD)	10 (0)	10.43 (5.71)	10 (6.16)
	median (min, max)	10 (10, 10)	10 (4, 20)	10 (4, 20)
How many cigarettes do you smoke per day? (categorial variable-smokers only)	10 or less	8 (73%)	14 (64%)	11 (69%)
	11-20	3 (27%)	8 (36%)	5 (31%)
	Missing	19	37	30
How soon do you have your first smoke?	don’t smoke at all			
	Within 5 minutes	1 (10%)	7 (35%)	4 (29%)
	6-30 minutes	4 (40%)	5 (25%)	2 (14%)
	31-60 minutes	3 (30%)	4 (20%)	2 (14%)
	Over 60 minutes	2 (20%)	4 (20%)	6 (43%)
	Missing	20	39	32
Heaviness of Smoking Index	n	10	20	14
	mean (SD)	4.7 (1.06)	5.15 (1.46)	4.64 (1.65)
	median (min, max)	4.5 (3, 6)	5 (3, 7)	4 (3, 7)
Have you ever tried to quit smoking?(Yes/No. Smokers only)	No, I haven't tried to quit		2 (9.1%)	1 (6.3%)
	Yes, I've tried to quit	11 (100%)	20 (91%)	15 (94%)
	Missing	19	37	30

Supplementary Table 7: ***Characteristics of participants that use the Internet***

Question	Option	To Quit Smoking (n=49)	Your Health (n=67)	For a Baby (n=57)
What is your age?	n	49	66	56
	mean (SD)	36.63 (12.72)	35.58 (11.59)	36.16 (11.9)
	median (min, max)	38 (0, 60)	34 (0, 59)	34 (16, 64)
Dichotomised Age	16-49 years	38 (79%)	55 (85%)	46 (82%)
	50+ years	10 (21%)	10 (15%)	10 (18%)
	Missing	1	2	1
Do you identify as Aboriginal or Torres Strait Islander?	Yes, Aboriginal	46 (94%)	64 (96%)	53 (95%)
	Yes, Aboriginal & Torres Strait Islander	3 (6.1%)	3 (4.5%)	3 (5.4%)
	Missing	0	0	1
Are you currently pregnant?	No	46 (96%)	61 (92%)	51 (91%)
	Yes	2 (4.2%)	5 (7.6%)	5 (8.9%)
	Missing	1	1	1
How far into your pregnancy are you?(if pregnant)	up to 12 weeks			
	13 to 24 weeks	1 (50%)	1 (20%)	1 (20%)
	25 to 40 weeks	1 (50%)	4 (80%)	4 (80%)
	Missing	47	62	52
Have you given birth before?(if not pregnant)	No	5 (14%)	10 (22%)	8 (21%)
	Yes	30 (86%)	35 (78%)	30 (79%)
	Missing	14	22	19
If yes, how many babies and/or children are living in your household?	No Children	14 (30%)	19 (30%)	13 (23%)
	1 child	4 (8.5%)	6 (9.4%)	9 (16%)
	2 children	10 (21%)	10 (16%)	9 (16%)
	3 children	11 (23%)	16 (25%)	12 (21%)
	4 or more children	8 (17%)	13 (20%)	13 (23%)
	Missing	2	3	1
Number of tobacco Smokers in household?	0	20 (41%)	29 (43%)	23 (40%)
	1	18 (37%)	20 (30%)	18 (32%)
	2-3	10 (20%)	17 (25%)	14 (25%)
	More than 3	1 (2.0%)	1 (1.5%)	2 (3.5%)
	Missing	0	0	0
How does your household manage places where smoking is allowed?	Complete ban on smoking anywhere inside	9 (20%)	12 (19%)	11 (21%)

	Smoking only on verandah/immediate area	34 (74%)	46 (73%)	39 (74%)
	Smoking allowed in some rooms & on veranda	2 (4.3%)	3 (4.8%)	2 (3.8%)
	People can smoke anywhere in or outside	1 (2.2%)	2 (3.2%)	1 (1.9%)
	Missing	3	4	4
Do you currently smoke tobacco?	Yes	20 (42%)	26 (39%)	24 (42%)
	No, I used to smoke tobacco but have quit	14 (29%)	19 (28%)	16 (28%)
	No, I have never smoked tobacco of any time	14 (29%)	22 (33%)	17 (30%)
	Missing	1	0	0
How often do you smoke tobacco?(smokers only)	Daily	12 (63%)	17 (71%)	16 (70%)
	Non-Daily	7 (37%)	7 (29%)	7 (30%)
	Missing	30	43	34
How many cigarettes do you smoke per day? (continuous variable-smokers only)	n	8	8	6
	mean (SD)	7.38 (2.88)	8.5 (3.85)	8.33 (2.66)
	median (min, max)	8 (4, 10)	10 (4, 15)	10 (4, 10)
How many cigarettes do you smoke per day? (categorial variable-smokers only)	10 or less	16 (80%)	17 (68%)	16 (67%)
	11-20	4 (20%)	8 (32%)	8 (33%)
	Missing	29	42	33
How soon do you have your first smoke?	don't smoke at all			
	Within 5 minutes	3 (17%)	7 (30%)	3 (14%)
	6-30 minutes	7 (39%)	8 (35%)	8 (36%)
	31-60 minutes	3 (17%)	4 (17%)	5 (23%)
	Over 60 minutes	5 (28%)	4 (17%)	6 (27%)
	Missing	31	44	35
Heaviness of Smoking Index	n	18	23	22
	mean (SD)	4.67 (1.33)	5.13 (1.42)	4.73 (1.35)
	median (min, max)	5 (3, 7)	5 (3, 7)	4.5 (3, 7)
Have you ever tried to quit smoking?(Yes/No. Smokers only)	No, I haven't tried to quit	2 (10%)	3 (12%)	1 (4.2%)
	Yes, I've tried to quit	18 (90%)	22 (88%)	23 (96%)
	Missing	29	42	33

Supplementary Table 8: ***Characteristics of participants that use an App***

Question	Option	To Quit Smoking (n=35)	Your Health (n=42)	For a Baby (n=37)
What is your age?	n	35	41	36
	mean (SD)	37.6 (12.5)	36.63 (10.51)	37.47 (11.96)
	median (min, max)	36 (16, 59)	34 (21, 58)	35 (17, 64)
Dichotomised Age	16-49 years	27 (77%)	34 (83%)	29 (81%)
	50+ years	8 (23%)	7 (17%)	7 (19%)
	Missing	0	1	1
Do you identify as Aboriginal or Torres Strait Islander?	Yes, Aboriginal	33 (94%)	38 (90%)	35 (95%)
	Yes, Aboriginal & Torres Strait Islander	2 (5.7%)	4 (9.5%)	2 (5.4%)
	Missing	0	0	0
Are you currently pregnant?	No	34 (100%)	38 (90%)	33 (92%)
	Yes		4 (9.5%)	3 (8.3%)
	Missing	1	0	1
How far into your pregnancy are you?(if pregnant)	up to 12 weeks			
	13 to 24 weeks			1 (33%)
	25 to 40 weeks		4 (100%)	2 (67%)
	Missing	35	38	34
Have you given birth before?(if not pregnant)	No	6 (20%)	3 (11%)	2 (9.5%)
	Yes	24 (80%)	25 (89%)	19 (90%)
	Missing	5	14	16
If yes, how many babies and/or children are living in your household?	No Children	10 (30%)	11 (26%)	7 (19%)
	1 child	2 (6.1%)	4 (9.5%)	5 (14%)
	2 children	9 (27%)	8 (19%)	7 (19%)
	3 children	9 (27%)	10 (24%)	10 (28%)
	4 or more children	3 (9.1%)	9 (21%)	7 (19%)
	Missing	2	0	1
Number of tobacco Smokers in household?	0	17 (49%)	20 (48%)	16 (43%)
	1	12 (34%)	10 (24%)	10 (27%)
	2-3	5 (14%)	11 (26%)	10 (27%)
	More than 3	1 (2.9%)	1 (2.4%)	1 (2.7%)
	Missing	0	0	0
How does your household manage places where smoking is allowed?	Complete ban on smoking anywhere inside	6 (18%)	10 (24%)	9 (25%)

	Smoking only on verandah/immediate area	24 (71%)	29 (69%)	23 (64%)
	Smoking allowed in some rooms & on veranda	3 (8.8%)	2 (4.8%)	3 (8.3%)
	People can smoke anywhere in or outside	1 (2.9%)	1 (2.4%)	1 (2.8%)
	Missing	1	0	1
Do you currently smoke tobacco?	Yes	14 (40%)	18 (43%)	15 (41%)
	No, I used to smoke tobacco but have quit	8 (23%)	7 (17%)	10 (27%)
	No, I have never smoked tobacco of any time	13 (37%)	17 (40%)	12 (32%)
	Missing	0	0	0
How often do you smoke tobacco?(smokers only)	Daily	11 (79%)	13 (72%)	11 (73%)
	Non-Daily	3 (21%)	5 (28%)	4 (27%)
	Missing	21	24	22
How many cigarettes do you smoke per day? (continuous variable-smokers only)	n	6	6	4
	mean (SD)	9.83 (3.49)	9.83 (3.49)	9 (2)
	median (min, max)	10 (4, 15)	10 (4, 15)	10 (6, 10)
How many cigarettes do you smoke per day? (categorial variable-smokers only)	10 or less	8 (57%)	11 (61%)	9 (60%)
	11-20	6 (43%)	7 (39%)	6 (40%)
	Missing	21	24	22
How soon do you have your first smoke?	don’t smoke at all			
	Within 5 minutes	3 (23%)	6 (35%)	3 (21%)
	6-30 minutes	6 (46%)	5 (29%)	4 (29%)
	31-60 minutes	2 (15%)	4 (24%)	3 (21%)
	Over 60 minutes	2 (15%)	2 (12%)	4 (29%)
	Missing	22	25	23
Heaviness of Smoking Index	n	13	17	14
	mean (SD)	5.23 (1.36)	5.29 (1.31)	4.86 (1.46)
	median (min, max)	6 (3, 7)	5 (3, 7)	4.5 (3, 7)
Have you ever tried to quit smoking?(Yes/No. Smokers only)	No, I haven't tried to quit	1 (7.1%)	3 (17%)	2 (13%)
	Yes, I've tried to quit	13 (93%)	15 (83%)	13 (87%)
	Missing	21	24	22

Supplementary Table 9: ***Characteristics of participants that use other supports***

Question	Option	To Quit Smoking (n=10)	Your Health (n=12)	For a Baby (n=15)
What is your age?	n	10	12	15
	mean (SD)	41.8 (14.51)	35.83 (9.8)	35.47 (12.62)
	median (min, max)	45.5 (21, 61)	35 (22, 52)	34 (17, 64)
Dichotomised Age	16-49 years	6 (60%)	10 (83%)	13 (87%)
	50+ years	4 (40%)	2 (17%)	2 (13%)
	Missing	0	0	0
Do you identify as Aboriginal or Torres Strait Islander?	Yes, Aboriginal	10 (100%)	12 (100%)	14 (100%)
	Yes, Aboriginal & Torres Strait Islander			
	Missing	0	0	1
Are you currently pregnant?	No	10 (100%)	11 (92%)	14 (93%)
	Yes		1 (8.3%)	1 (6.7%)
	Missing	0	0	0
How far into your pregnancy are you?(if pregnant)	up to 12 weeks			
	13 to 24 weeks			
	25 to 40 weeks		1 (100%)	1 (100%)
	Missing	10	11	14
Have you given birth before?(if not pregnant)	No	4 (44%)	2 (25%)	2 (25%)
	Yes	5 (56%)	6 (75%)	6 (75%)
	Missing	1	4	7
If yes, how many babies and/or children are living in your household?	No Children	4 (40%)		3 (20%)
	1 child		2 (17%)	3 (20%)
	2 children	1 (10%)	3 (25%)	1 (6.7%)
	3 children	2 (20%)	2 (17%)	3 (20%)
	4 or more children	3 (30%)	5 (42%)	5 (33%)
	Missing	0	0	0
Number of tobacco Smokers in household?	0		7 (58%)	8 (53%)
	1	6 (60%)	3 (25%)	3 (20%)
	2-3	2 (20%)	2 (17%)	4 (27%)
	More than 3	2 (20%)		
	Missing	0	0	0
How does your household manage places where smoking is allowed?	People can smoke anywhere in or outside			

	Complete ban on smoking anywhere inside	3 (30%)	4 (33%)	5 (33%)
	Smoking only on verandah/immediate area	7 (70%)	7 (58%)	10 (67%)
	Smoking allowed in some rooms & on veranda		1 (8.3%)	
	Missing	0	0	0
Do you currently smoke tobacco?	Yes	3 (30%)	4 (33%)	5 (33%)
	No, I used to smoke tobacco but have quit	1 (10%)	3 (25%)	3 (20%)
	No, I have never smoked tobacco of any time	6 (60%)	5 (42%)	7 (47%)
	Missing	0	0	0
How often do you smoke tobacco?(smokers only)	Daily	1 (33%)	2 (50%)	4 (80%)
	Non-Daily	2 (67%)	2 (50%)	1 (20%)
	Missing	7	8	10
How many cigarettes do you smoke per day? (continuous variable-smokers only)	n	2	2	1
	mean (SD)	10 (0)	8 (2.83)	10 (.)
	median (min, max)	10 (10, 10)	8 (6, 10)	10 (10, 10)
How many cigarettes do you smoke per day? (categorial variable-smokers only)	10 or less	3 (100%)	4 (100%)	4 (80%)
	11-20			1 (20%)
	Missing	7	8	10
How soon do you have your first smoke?	don't smoke at all	1 (50%)		
	Over 60 minutes			
	Within 5 minutes		1 (33%)	1 (25%)
	6-30 minutes	1 (50%)	1 (33%)	1 (25%)
	31-60 minutes		1 (33%)	2 (50%)
	Missing	8	9	11
Heaviness of Smoking Index	n	2	3	4
	mean (SD)	5.5 (2.12)	4 (1)	5 (1.41)
	median (min, max)	5.5 (4, 7)	4 (3, 5)	4.5 (4, 7)
Have you ever tried to quit smoking?(Yes/No. Smokers only)	No, I haven't tried to quit		1 (25%)	1 (20%)
	Yes, I've tried to quit	3 (100%)	3 (75%)	4 (80%)
	Missing	7	8	10

Supplementary Table 10: ***Characteristics of participants that use quit medications***

Question	Option	To Quit Smoking (n=64)
What is your age?	n	62
	mean (SD)	37.95 (13.11)
	median (min, max)	38 (0, 61)
Dichotomised Age	16-49 years	46 (75%)
	50+ years	15 (25%)
	Missing	3
Do you identify as Aboriginal or Torres Strait Islander?	Yes, Aboriginal	59 (94%)
	Yes, Aboriginal & Torres Strait Islander	4 (6.3%)
	Missing	1
Are you currently pregnant?	No	58 (92%)
	Yes	5 (7.9%)
	Missing	1
How far into your pregnancy are you?(if pregnant)	up to 12 weeks	
	13 to 24 weeks	1 (20%)
	25 to 40 weeks	4 (80%)
	Missing	59
Have you given birth before?(if not pregnant)	No	10 (20%)
	Yes	39 (80%)
	Missing	15
If yes, how many babies and/or children are living in your household?	No Children	25 (40%)
	1 child	2 (3.2%)
	2 children	11 (18%)
	3 children	16 (26%)
	4 or more children	8 (13%)
	Missing	2
Number of tobacco Smokers in household?	0	27 (43%)
	1	19 (30%)
	2-3	16 (25%)
	More than 3	1 (1.6%)
	Missing	1
How does your household manage places where smoking is allowed?	Complete ban on smoking anywhere inside	11 (18%)
	Smoking only on verandah/immediate area	44 (71%)
	Smoking allowed in some rooms & on veranda	6 (9.7%)
	People can smoke anywhere in or outside	1 (1.6%)
	Missing	2

Do you currently smoke tobacco?	Yes	28 (44%)
	No, I used to smoke tobacco but have quit	15 (24%)
	No, I have never smoked tobacco of any time	20 (32%)
	Missing	1
How often do you smoke tobacco?(smokers only)	Daily	23 (82%)
	Non-Daily	5 (18%)
	Missing	36
How many cigarettes do you smoke per day? (continuous variable-smokers only)	n	12
	mean (SD)	10.25 (5.19)
	median (min, max)	10 (4, 20)
How many cigarettes do you smoke per day? (categorial variable-smokers only)	10 or less	16 (57%)
	11-20	12 (43%)
	Missing	36
How soon do you have your first smoke?	don't smoke at all	
	Within 5 minutes	7 (26%)
	6-30 minutes	12 (44%)
	31-60 minutes	6 (22%)
	Over 60 minutes	2 (7.4%)
	Missing	37
Heaviness of Smoking Index	n	27
	mean (SD)	5.33 (1.21)
	median (min, max)	5 (3, 7)
Have you ever tried to quit smoking?(Yes/ No. Smokers only)	No, I haven't tried to quit	4 (14%)
	Yes, I've tried to quit	24 (86%)
	Missing	36
